# Supplementary figures and images for: Quantification of DNA of Fusarium culmorum and Trichothecene Genotypes 3ADON and NIV in the Grain of Winter Wheat
Source: Pathogens. 2022 Nov 30;11(12):1449. doi: 10.3390/pathogens11121449 (PMC9788549; doi:10.3390/pathogens11121449)

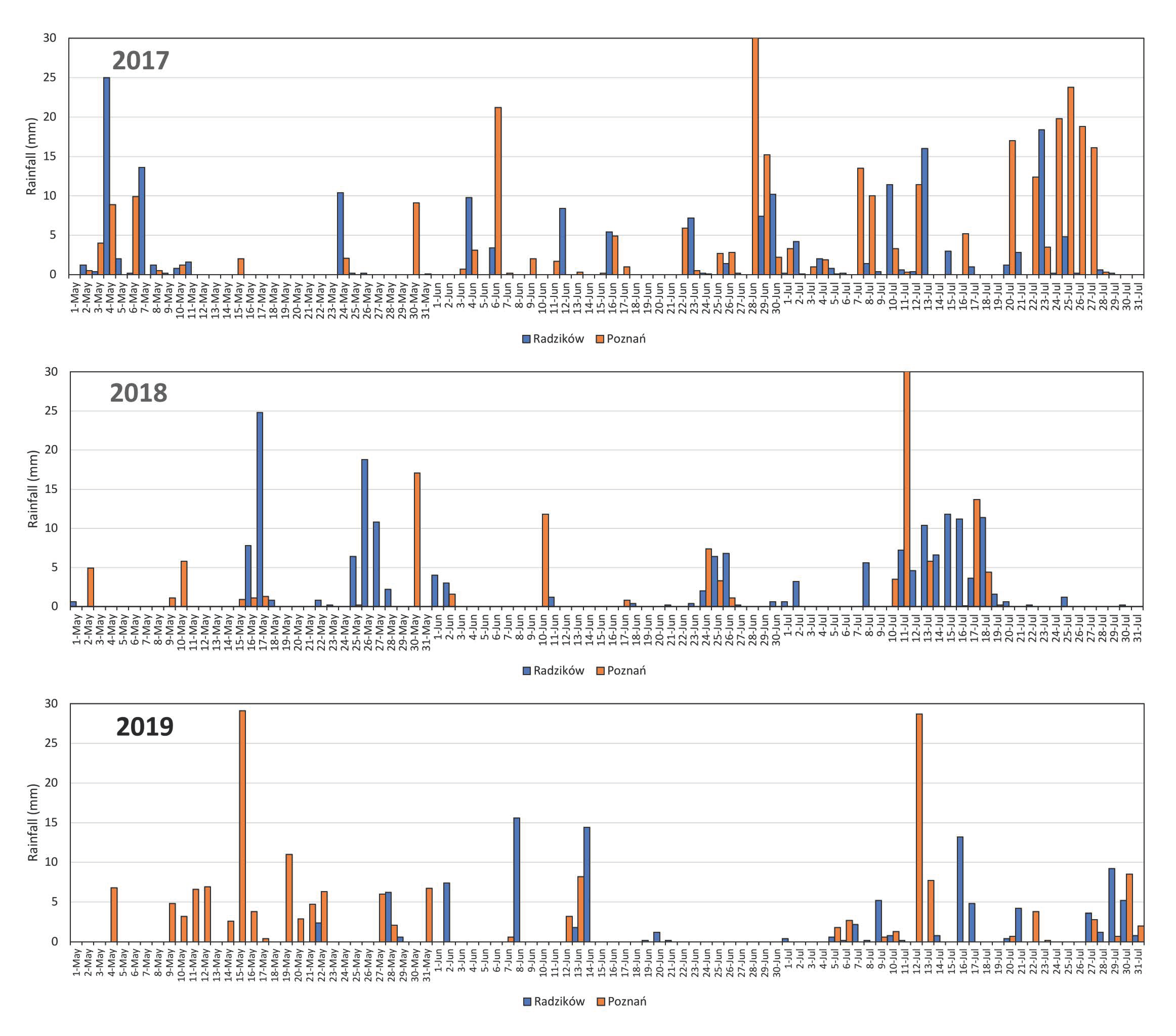

Supplement: Supplementary file 1 [file pathogens-11-01449-s001.zip › pathogens-2064597-supplementary.tif]
